# Physiological arterial pressure improves renal performance during normothermic machine perfusion in a porcine kidney DCD model

**DOI:** 10.1016/j.heliyon.2024.e41610

**Published:** 2025-01-10

**Authors:** Yitian Fang, Gisela Ambagtsheer, Lin Xia, Marian C. Clahsen-van Groningen, Robert C. Minnee, Ron W.F. de Bruin

**Affiliations:** a Division of HPB and Transplant Surgery, Department of Surgery, Transplant Institute, Erasmus Medical Center, Rotterdam, the Netherlands; b Department of Ophthalmology, First Affiliated Hospital of Anhui Medical University, Hefei, China; c Department of Pathology and Clinical Bioinformatics, Erasmus Medical Center, Rotterdam, the Netherlands; d Institute of Experimental and Systems Biology, RWTH Aachen University, Aachen, Germany

## Abstract

**Background:**

Normothermic machine perfusion (NMP) provides a platform for kidney quality assessment. Donation after circulatory death (DCD) donor kidneys are associated with great ischemic injury and high intrarenal resistance (IRR). This experimental study aims to investigate the impact of different perfusion pressures on marginal kidney function and injury during NMP.

**Methods:**

Twenty-seven slaughterhouse porcine kidneys were retrieved and subjected to 60 min of warm ischemia time to mimic DCD condition. These kidneys were randomized into 75 mmHg (subphysiological, n = 9), 95 mmHg (physiological, n = 9), and 115 mmHg NMP (high physiological, n = 9). Renal function and injury were assessed during NMP.

**Results:**

Three groups showed comparable IRR, with the 115 mmHg group exhibiting the highest blood flow. The 95 mmHg group [0.48 (0.36–1.15) ml/min/100g] and 115 mmHg group [0.93 (0.45–1.41) ml/min/100g] showed significantly higher creatinine clearance compared to the 75 mmHg group [0.16 (0.08–0.37) ml/min/100g] during the first hour of NMP (p = 0.049, p = 0.009, respectively). The 115 mmHg group exhibited significantly higher oxygen consumption compared to the 75 mmHg group at 30 min of NMP [1.37 (1.05–1.92) versus 0.72 (0.61–0.82) mlO_2_/min/100g, p = 0.009]. Perfusate neutrophil gelatinase-associated lipocalin (NGAL) levels were consistently lowest in the 95 mmHg group and highest in the 75 mmHg group. Aspartate aminotransferase (AST) levels of the 115 mmHg group were significantly higher than the 75 mmHg group.

**Conclusions:**

For kidneys with high IRR, both 95 mmHg and 115 mmHg perfusion pressures enable an early improvement in renal hemodynamics and function compared to 75 mmHg during NMP, while a high physiological perfusion can cause additional injury.

## Abbreviations

AST: aspartate aminotransferase
ATN: acute tubular necrosis
ATP: adenosine triphosphate
CrCl: creatinine clearance
DCD: donation after circulatory death
FENa: fractional excretion of sodium
IQR: interquartile range
IRR: intrarenal resistance
MAP: mean arterial pressure
NGAL: neutrophil gelatinase-associated lipocalin
NMP: normothermic machine perfusion
PAS: periodic acid-Schiff
RBF: renal blood flow
SD: standard deviation
TNa: total sodium reabsorption
VO_2_: oxygen consumption
WIT: warm ischemia time

## 1 Introduction

Selecting eligible organs from marginal donors, such as those from donation after circulatory death (DCD) donors, poses a challenge in the field of kidney transplantation [1,2]. Normothermic machine perfusion (NMP) has emerged as a promising platform for kidney quality assessment before transplantation by recreating a physiological environment conducive to organ restoration and energy substrate replenishment.

DCD donor kidneys are strongly associated with substantial ischemic injury and increased risk of graft failure due to prolonged non-heart-beating periods. Warm ischemia, characteristic of DCD donation, results in rapid depletion of intracellular adenosine triphosphate (ATP), thereby disrupting ATP-dependent cellular processes. This disruption includes the inhibition of Na^+^-K^+^-ATPase activity, leading to intracellular sodium accumulation and subsequent cell swelling, particularly within the renal medulla [3,4]. This intracellular swelling increases the osmotic gradient, driving water into the mitochondrial matrix and causing matrix swelling [5]. Consequently, DCD donor kidneys frequently exhibit high intrarenal resistance (IRR) and inferior renal function during NMP.

Increasing arterial perfusion pressure has been proven an effective strategy to improve renal perfusion and function during NMP [6,7]. However, the appropriate pressure to ensure sufficient blood flow without causing additional vascular injury for such kidneys remains unclear.

Previous studies have mostly perfused kidneys at mean arterial pressures ranging from 50 to 85 mmHg, both in experimental and clinical settings [[8], [9], [10], [11]]. Such subphysiological pressures may not be optimally tailored to address the compromised intravascular condition of marginal kidneys. Research has shown that kidneys perfused at 95 mmHg demonstrated superior renal function without additional endothelial damage compared to those perfused at 75 mmHg [7]. However, this study did not explore the impact of such physiological pressure on marginal kidneys and its subsequent effect on tubular injury, where the ischemic damage primarily occurs.

This study aims to fill this gap by investigating the impact of subphysiological, physiological, and high physiological arterial perfusion pressures on renal function and injury in a porcine kidney DCD model characterized by high IRR.

## 2 Materials and methods

### 2.1 Study design

Twenty-seven porcine kidneys were procured from juvenile female landrace pigs (weighing ∼100 kg) in a local slaughterhouse. We chose this organ source due to its comparable dimensions to human kidneys and ease of obtainment [12]. These kidneys were randomized into three groups with varying perfusion pressures: subphysiological (75 mmHg, n = 9), physiological (95 mmHg, n = 9), and high physiological (115 mmHg, n = 9). To simulate the conditions of DCD donor kidneys, each kidney was subjected to 60 min of warm ischemia time (WIT) to induce comparable ischemic injury and high IRR. Subsequently, all kidneys underwent NMP for 2 h, during which renal function and injury were assessed at designated time points. The 2-h NMP duration was chosen based on current clinical NMP practices, where one to 2 h of perfusion are commonly used [10,13].

This study was conducted in compliance with the European Communities Council Directive 2010/63/EU and the 3R principle. By utilizing kidneys from pigs slaughtered for meat production, we reduced the need for additional animal sacrifice for research purposes. Since the kidneys were obtained from pigs slaughtered for meat consumption, no approval from the Medical Ethics Committee was required.

### 2.2 Organ retrieval

After the pig was stunned, 2 L of autologous blood were collected in a bucket containing 25000 IU of heparin (LEO Pharma A/S, Ballerup, Denmark). Following kidney retrieval, each organ was subjected to 60 min WIT, arterially cannulated, and flushed with 500 ml cold Ringer's lactate (Baxter BV, Utrecht, the Netherlands). Kidneys were then transported to the laboratory using hypothermic machine perfusion (LifePort→, Organ Recovery Systems, Itasca, USA) with 1 L of University of Wisconsin Belzer machine perfusion solution (Belzer MPS®) at a pressure of 30 mmHg until NMP. The duration from kidney retrieval to NMP ranged from 2 to 7 h. Throughout this period, the kidneys remained under hypothermic machine perfusion. As a standard preservation method in the Netherlands for deceased donor kidneys, its impact on renal function and injury is expected to be minimal [14]. The collected blood was processed to deplete leukocytes using a leukocyte reduction filter (BioR-plus, Fresenius Kabi AG, Homburg, Germany) and stored at 4 °C for further use.

### 2.3 Normothermic machine perfusion

All kidneys were perfused in the laboratory using the NMP setup commercially available from Harvard Apparatus®, Germany, as previously described [15]. In brief, kidneys were perfused with an oxygenated, red blood cell-based solution at 37 °C in a pressure-controlled mode, achieving the settled pressure by adjusting the centrifugal pump speed. The detailed components of the perfusate is provided in Table S1. The urine output was recorded every 30 min and added back to the perfusate after measurement. This recirculation was done to maintain electrolyte balance and ensure a constant circulating volume within the perfusion system. Creatinine was added in the perfusate to evaluate renal clearance.

### 2.4 Renal function and injury assessment

Throughout NMP, renal blood flow (RBF) was continuously monitored and urine output was measured every 30 min. Perfusate and urine samples were collected at 30-min intervals for further analysis. Oxygen consumption (VO_2_) and perfusate lactate levels were determined through blood gas analyses. Creatinine clearance (CrCl), fractional excretion of sodium (FENa) and total sodium reabsorption (TNa) were calculated using established equations [16,17].

To assess tubular injury under different perfusion pressures, neutrophil gelatinase-associated lipocalin (NGAL) was quantified in the perfusate as an early biomarker of tubular injury using a pig NGAL ELISA kit (BioPorto Diagnostics A/S, Hellerup, Denmark). Perfusate samples were also analyzed at the clinical biochemistry lab (Triallab, Erasmus Medical Center) to determine lactate dehydrogenase (LDH) and aspartate aminotransferase (AST) levels.

### 2.5 Histopathology assessment

Renal cortex biopsies were taken from each kidney before and after NMP, fixed in 4 % buffered paraformaldehyde, and embedded in paraffin before being sectioned into 5 μm slices. Using periodic acid-Schiff (PAS) staining, acute tubular necrosis (ATN) and acute glomerular ischemia were graded on a scale of 0–3 (0-none, 1-mild, 2-moderate, 3-severe injury) by an expert renal pathologist (M.C.v.G) blinded to the study. ATN severity was determined based on brush border loss, tubular dilatation, tubular cell necrosis, vacuolation, and interstitial edema at the cortical-medullary junction. Acute glomerular ischemia was assessed by Bowman's space enlargement and glomerular capillary collapse.

### 2.6 Statistical analysis

Continuous variables are presented as mean ± standard deviation (SD) if normally distributed or median with interquartile range (IQR) in the case of non-normal distribution. Two-way ANOVA was used to compare the functional and viability markers between groups during NMP. Fixed effects were time, group factor, and the interaction of the group factor with time, while individual kidneys were considered as random effects. A Geisser–Greenhouse correction and a restricted maximum likelihood approach were used. A p-value of ≤0.05 was considered statistically signiﬁcant. Statistical analysis was performed using GraphPad Prism 9.3.1 (GraphPad Software Inc., San Diego, CA).

## 3 Results

The median cold ischemia time was 5.9 (3.3–6.1), 3.8 (2.8–6.3), 4.4 (3.0–6.0) hours, and the median weight was 262 (254–298), 261 (232–273), 264 (252–284) grams in the 75 mmHg, 95 mmHg, and 115 mmHg groups, respectively (p = 0.755 and p = 0.951, respectively). All kidneys underwent NMP for 2 h without macroscopic abnormalities.

### 3.1 Hemodynamics

**Fig. 1:**
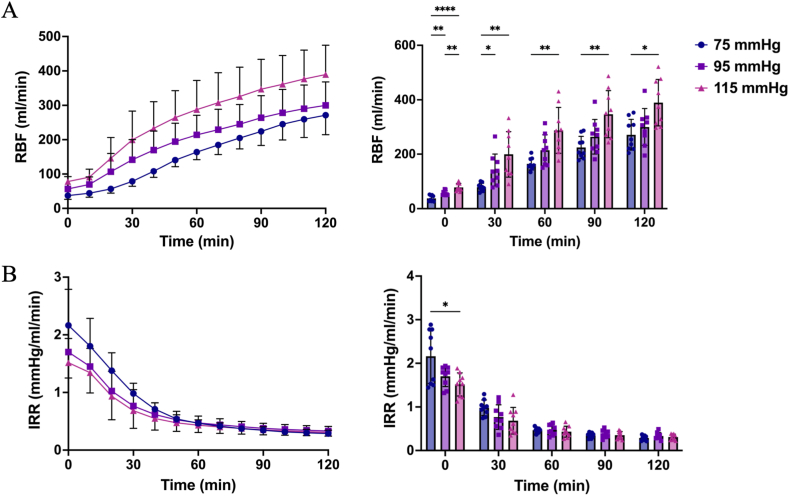
(A) Renal blood flow (RBF), and (B) intrarenal resistance (IRR) during normothermic machine perfusion (NMP). Data presented as median ± SD. ∗p < 0.05, ∗∗p < 0.01, ∗∗∗p < 0.001, ∗∗∗∗p < 0.0001.

RBF exhibited a sharp increase over time in all groups during NMP (p < 0.001). In the first 30 min, RBF in the 75 mmHg group was significantly lower compared to the 95 mmHg and 115 mmHg groups (p = 0.029 and p = 0.006, respectively). This difference between the 75 mmHg and 95 mmHg groups diminished with perfusion ongoing (p = 0.08), but RBF remained significantly higher in the 115 mmHg group compared to the 75 mmHg group (p = 0.010). At the end of perfusion, the mean RBF was 272 ± 57, 300 ± 68, and 389 ± 85 ml/min for the 75 mmHg, 95 mmHg, and 115 mmHg groups, respectively (Fig. 1A).

IRR decreased significantly in all three groups over time (p < 0.001). Higher perfusion pressures were associated with lower IRR during the first hour of NMP. During the second hour, intergroup differences diminished, with IRR maintaining around 0.31 mmHg/ml/min in all groups (p = 0.387, Fig. 1B).

### 3.2 Renal function

**Fig. 2:**
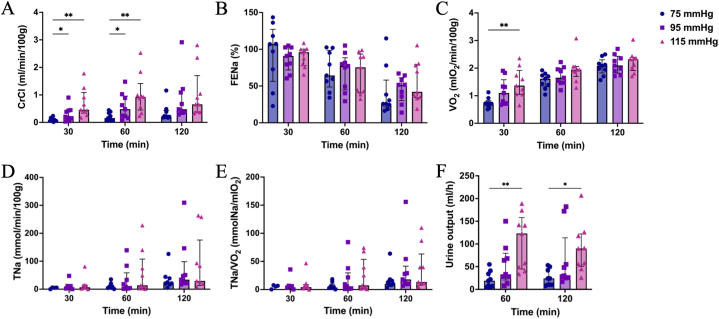
Renal function assessment during NMP. (A) creatinine clearance (CrCl, ml/min/100g); (B) fractional excretion of sodium (FENa, %); (C) oxygen consumption (VO_2_, mlO_2_/min/100g); (D) total sodium reabsorption (TNa, mmol/min/100g); (E) oxygen utilization efficiency (TNa/VO_2_, mmolNa/mlO_2_); (F) urine output. Data are presented as median with interquartile range. ∗p < 0.05, ∗∗p < 0.01, ∗∗∗p < 0.001.

There was a statistically significant difference in CrCl when comparing the 75 mmHg group to the 95 mmHg and 115 mmHg groups during the first hour of NMP [median (IQR), 75 mmHg 0.16 (0.08–0.37) versus 95 mmHg 0.48 (0.36–1.15) ml/min/100g, p = 0.049; 75 mmHg 0.16 (0.08–0.37) versus 115 mmHg 0.93 (0.45–1.41) ml/min/100g, p = 0.009]. This trend persisted during the second hour, although it lost its significance, with median CrCl of 0.21 (0.18–0.44), 0.48 (0.36–1.15), 0.66 (0.39–1.70) ml/min/100g at the end of NMP (Fig. 2A). FENa decreased significantly over time in all three groups (p < 0.001). There was no significant difference between the groups at the end of NMP [75 mmHg 25.7 (21.5–58.1)%, 95 mmHg 51.6 (30.2–61.3)%, 115 mmHg 42.1 (34.1–79.3)%, p = 0.529, Fig. 2B]. VO_2_ increased over time in all three groups (p < 0.001). At 30 min of NMP, the 115 mmHg group exhibited significantly higher VO_2_ compared to the 75 mmHg group [75 mmHg 0.72 (0.61–0.82) versus 115 mmHg 1.37 (1.05–1.92) mlO_2_/min/100g, p = 0.009]. However, VO_2_ in the 75 mmHg group increased rapidly, reaching a comparable level with the other two groups by the end of NMP [75 mmHg 2.08 (1.82–2.30), 95 mmHg 2.10 (1.82–2.43), 115 mmHg 2.32 (1.91–2.39) mlO_2_/min/100g, p = 0.544, Fig. 2C].

Furthermore, higher perfusion pressure contributed to higher TNa, although not statistically significant between the three groups [75 mmHg 21.09 (11.29–41.47), 95 mmHg 34.03 (21.74–98.51), 115 mmHg 29.62 (13.67–175.81) mmol/min/100g, p = 0.376, Fig. 2D]. Similarly, oxygen utilization followed the same trend as TNa, with no significant difference between groups at the end of NMP [75 mmHg 9.95 (5.54–16.82), 95 mmHg 17.96 (9.99–41.70), 115 mmHg 13.52 (7.87–63.30) mmolNa/mlO_2_, p = 0.352, Fig. 2E]. Both kidneys in the 95 mmHg and 115 mmHg groups produced more urine than those in the 75 mmHg group, although this was statistically significant only between the 75 mmHg and 115 mmHg groups during the 2-h NMP [75 mmHg 24 (10–46) versus 95 mmHg 30 (26–114) ml/h, p = 0.200; 75 mmHg 24 (10–46) versus 115 mmHg 90 (51–122) ml/h, p = 0.015, Fig. 2F].

### 3.3 Renal injury

**Fig. 3:**
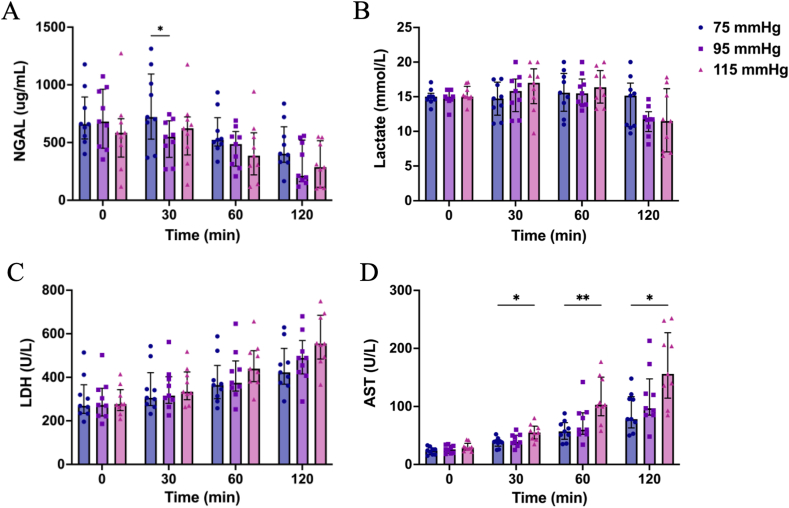
Renal injury assessment during NMP. (A) Neutrophil gelatinase-associated lipocalin (NGAL, ug/ml); (B) perfusate lactate (mmol/L); (C) lactate dehydrogenase (LDH, U/L) and (D) aspartate aminotransferase (AST, U/L). Data are presented as median with interquartile range. ∗p < 0.05, ∗∗p < 0.01.

Baseline NGAL levels were comparable in the three groups [75 mmHg 659.7 (531.2–894.4), 95 mmHg 682.3 (451.0–961.8), 115 mmHg 584.7 (374.7–706.9) μg/ml, p = 0.558]. In both the 75 mmHg and 115 mmHg groups, NGAL levels exhibited a trend of initial increase followed by a decrease, while in the 95 mmHg group, NGAL levels decreased continuously, with median values of 403.3 (329.5–637.0), 212.8 (162.5–521.3), and 286.8 (112.4–515.7) μg/ml at the end of NMP. At 30 min of NMP, NGAL levels were significantly lower in the 95 mmHg group compared to the 75 mmHg group [549.8 (372.3–689.0) vs. 721.6 (529.3–1094.5) μg/ml, p = 0.041, Fig. 3A]. Baseline perfusate lactate levels were similar in the three groups [75 mmHg 15.0 (14.5–15.5), 95 mmHg 15.0 (14.5–16.0), 115 mmHg 15.0 (14.9–16.5) mmol/L, p = 0.496], which slightly increased to 15.2 (10.7–17.0) mmol/L in the 75 mmHg group while decreasing to 11.7 (10.0–12.8) mmol/L in the 95 mmHg group and 11.5 (7.0–16.2) mmol/L in the 115 mmHg group at the end of NMP (Fig. 3B).

Both LDH and AST exhibited an increasing trend over time in all three groups. LDH levels were slightly higher in the 115 mmHg group compared to the other two groups [75 mmHg 423 (369–533), 95 mmHg 489 (416–569), 115 mmHg 555 (484–685) U/L, p = 0.075, Fig. 3C]. AST levels were slightly lower in the 75 mmHg group compared to the 95 mmHg [78 (63–117) versus 97 (85–148) U/L, p = 0.494], but significantly lower when compared to the 115 mmHg group throughout NMP (p = 0.002, Fig. 3D).

**Fig. 4:**
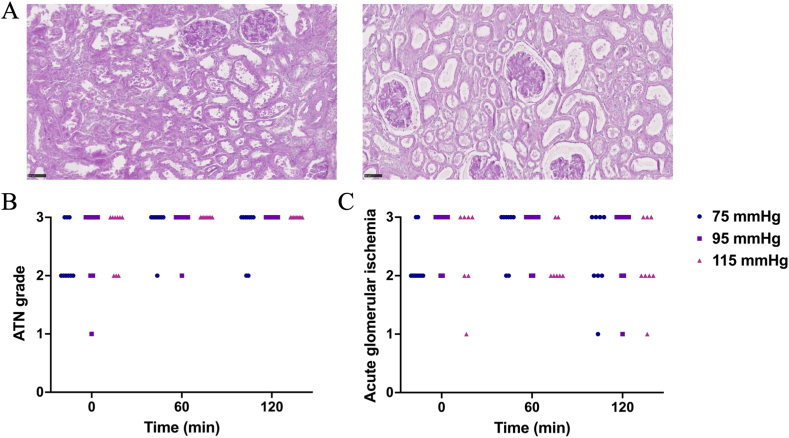
(A) Representative morphological images showing moderate acute tubular necrosis (ATN) and mild acute glomerular ischemia (left), and severe ATN and severe acute glomerular ischemia (right), respectively (PAS staining, 40 × magnification); (B) histological grades of ATN; (C) histological grades of acute glomerular ischemia.

Representative images of ATN and acute glomerular ischemia at moderate and severe levels are depicted in Fig. 4A. In all groups, most kidneys exhibited moderate to severe ATN. Throughout NMP, kidneys remained unchanged or slightly worsened in ATN, with no significant differences observed between the groups (p = 0.115, Fig. 4B). Similarly, most kidneys displayed moderate to severe acute glomerular ischemia, which remained unchanged or mildly worsened during NMP. No significant intergroup differences were observed (p = 0.903, Fig. 4C).

## 4 Discussion

In this study, we investigated the impact of different perfusion pressures (subphysiological, physiological, and high physiological) on the renal function and injury using a porcine kidney DCD model during NMP. Our findings indicate that compared to the commonly used perfusion pressure of 75 mmHg, a physiological perfusion pressure of 95 mmHg led to improved renal function without causing additional renal injury. However, NMP under a high physiological pressure (115 mmHg) led to additional injury despite achieving superior renal function. Increasing perfusion pressure to the physiological level could potentially salvage kidneys considered functionally insufficient under subphysiological perfusion pressures during NMP.

NMP represents a promising approach for assessing organ quality by providing oxygenated blood-based perfusate at normothermic temperatures, allowing kidneys to restore metabolism and exhibit functionality. By monitoring functional parameters during perfusion, it becomes feasible to identify marginal kidneys that are suitable for transplantation, thereby increasing the utilization of donor organs. For example, Hosgood et al. reported that 5 out of 10 initially declined kidneys were successfully transplanted based on renal blood flow and urine production during NMP at a perfusion pressure of 70 mmHg [18]. However, it could be argued that a more physiological perfusion pressure might enhance the functionality of the remaining kidneys, thereby meeting the transplantation criteria. Therefore, demonstrating a kidney's physiological function during NMP is crucial for optimizing organ selection and transplantation outcomes.

In clinical practice, increasing the mean arterial pressure (MAP) is commonly applied to achieve satisfactory reperfusion during kidney transplantation [[19], [20], [21]]. Under physiological conditions, renal artery pressure is naturally higher than normal blood pressure due to gravity. However, in most clinical studies, a subphysiological pressure of 75 mmHg has been chosen as the perfusion pressure for two reasons [10,13]. Firstly, the optimal perfusion pressure is still under exploration, and using a relatively low pressure serves as a cautious approach to preserve vascular integrity [22]. Secondly, it has been demonstrated to be safe to perfuse the kidneys at 75 mmHg, although most favorable outcome data come from kidneys initially considered eligible for transplantation, which typically exhibit low IRR and mild injury. Therefore, existing literature suggests the feasibility for NMP at 75 mmHg in “good” kidneys. However, for “poor” kidneys, it remains uncertain whether this perfusion pressure is optimal or if NMP under such pressure can facilitate the kidney to present optimal functionality.

In research settings, high perfusion pressures have been explored in a few preclinical studies. For example, NMP has successfully been performed on discarded kidneys for 24–48 h at 70–100 mmHg (MAP 90 mmHg) [9,23]. Similarly, Pool et al. [24] used porcine kidneys undergoing NMP for 7 h at a pressure of 110/70 mmHg (MAP 97 mmHg).

Our results demonstrated a trend towards better functionality and viability of kidneys perfused at higher perfusion pressures, particularly significant during the first 30 min of NMP. Consistent with previous literature, we observed higher RBF and lower IRR attributed associated with higher arterial perfusion pressure in the early stage of NMP [6,7,25]. During this phase, the kidneys experienced a dramatic transition from cold storage to normothermic perfusion, resulting in a rapid shift in metabolic demand. Higher arterial perfusion pressure ensured that sufficient effective blood volume reached the renal cortex, supporting ATP-dependent metabolic activity, as reflected by higher VO_2_. Additionally, renal functionality, indicated by CrCl, increased correspondingly with the elevated metabolic levels.

Interestingly, the significant differences in hemodynamics and renal functionality during the early stage of NMP diminished as perfusion progressed. The observed "catch-up" effect in the 75 mmHg group during the second hour of NMP, characterized by a rapid increase in RBF and VO_2_, suggests that kidneys perfused under subphysiological pressure exhibited autoregulatory mechanisms to optimize blood flow distribution. However, this “catch-up” effect is limited, as evidenced by relatively lower urine output and renal function at the end of NMP. To alleviate initial ischemia injury, applying enough perfusion pressure to provide sufficient blood volume is critical in the beginning of NMP.

We further assessed renal injury during NMP. NGAL release represents an intrinsic response of proximal tubule cells to ischemic injury [26]. The short half-life (10 min) makes it a sensitive and early marker of acute kidney injury [27]. All groups exhibited a decrease in perfusate NGAL compared to baseline levels, which is in contrast to previous studies demonstrating a significant increase in NGAL levels during prolonged NMP [23,28]. This discrepancy indicates that short-term NMP under physiological or high physiological pressures may not induce additional tubular injury. Moreover, kidneys perfused at 95 mmHg exhibited significantly lower NGAL levels at 30 min of NMP in comparison with the 75 mmHg group, suggesting a potential protective effect of physiological pressure NMP against ischemic injury in the initial stage. This finding complements previous research demonstrating that 95 mmHg NMP does not induce additional endothelial or glycocalyx injury compared to 75 mmHg NMP [7,25].

The alterations in perfusate lactate levels further support this point. Specifically, the lactate level in the 75 mmHg group was higher than its baseline level and the other two groups by the end of NMP. This finding is in accordance with previous literature indicating that higher perfusate lactate levels are correlated with lower blood flow and higher NGAL levels, making it a surrogate biomarker for the donor kidney function [29]. Lactate levels are influenced by both glycolysis and gluconeogenesis in the kidney [[30], [31], [32]]. The lactate accumulation in the 75 mmHg group may be attributed to limited blood flow and oxygen supply, which leads to low oxygen tension in the inner medulla, resulting in impaired lactate metabolism and anaerobic glycolysis [33].

The general injury biomarkers LDH and AST exhibited an upward trend during the perfusion process, indicating a general kidney injury increasement. This finding was confirmed by the histological results, which revealed signs of acute tubular necrosis and collapse of glomerular capillaries. Similar trends have been reported in other studies [9,24,25]. With the current perfusion techniques, ischemia-reperfusion injury may be inevitable during NMP. Interestingly, kidneys in the 75 mmHg and 95 mmHg groups exhibited comparable renal injury in terms of AST and LDH, while higher injury levels were observed in the 115 mmHg group. Considering 115 mmHg as a value of high physiological pressure, it may lead to renal cellular injury, resulting in the leakage of intracellular LDH and AST [34]. However, it is important to note that AST can also be released from damaged red blood cells. At higher flow rates and pump speeds, the mechanical stress may exacerbate red blood cell damage, resulting in increased AST release in the 115 mmHg group.

Our study has several limitations to be addressed. Firstly, although pig kidneys are recognized to have similar anatomical and metabolic characteristics to human kidneys, the use of a slaughterhouse porcine kidney model may not perfectly replicate the complexities of human renal physiology and pathology [35]. Secondly, our study primarily focused on markers of renal tubular injury. Additional injury markers, such as reactive oxygen species and endothelial cell injury markers such as CD31 and CD34, could provide a more comprehensive understanding of kidney injury during NMP. Thirdly, as a preliminary study, it is not sufficient to drive changes in clinical practice. Further research with longer perfusion duration and transplantation is needed to validate these findings.

## 5 Conclusion

For kidneys with high IRR, both 95 mmHg and 115 mmHg arterial perfusion pressures enable an early improvement in renal hemodynamics and function during NMP. However, a high physiological perfusion pressure can lead to additional injury, suggesting a delicate balance between renal function and injury in marginal kidneys.

## CRediT authorship contribution statement

**Yitian Fang:** Writing – original draft, Methodology, Investigation, Formal analysis, Data curation, Conceptualization. **Gisela Ambagtsheer:** Writing – review & editing, Methodology, Investigation, Data curation. **Lin Xia:** Writing – review & editing, Methodology. **Marian C. Clahsen-van Groningen:** Writing – review & editing, Investigation. **Robert C. Minnee:** Writing – review & editing, Investigation, Conceptualization. **Ron W.F. de Bruin:** Writing – review & editing, Supervision, Methodology, Conceptualization.

## Funding

None.

## Declaration of competing interest

The authors declare that they have no known competing financial interests or personal relationships that could have appeared to influence the work reported in this paper.
