# Supplementary material for: Physiological arterial pressure improves renal performance during normothermic machine perfusion in a porcine kidney DCD model
Source: Heliyon. 2025 Jan 10;11(2):e41610. doi: 10.1016/j.heliyon.2024.e41610 (PMC11773052; doi:10.1016/j.heliyon.2024.e41610)
Supplement: Multimedia component 1 [file mmc1.docx]

**Supplementary**

**Table S1. Components of the normothermic machine perfusion perfusate**

| **Priming** | **Dosage** |
| --- | --- |
| Ringer’s lactate (Baxter, The Netherlands) | 300 mL |
| Autologous leukocyte-depleted blood | 500 mL |
| **Additives** |  |
| Albumin (Prothya Biosolutions, the Netherlands) | 20 g |
| 8.4% sodium bicarbonate (B. Braun, Germany) | 10 mL |
| 5% glucose (Baxter, The Netherlands) | 10 mL |
| Verapamil (Centrafarm, the Netherlands) | 2.5 mg |
| Dexamethasone (Centrafarm, The Netherlands) | 6 mg |
| Creatinine (Sigma-Aldrich, the Netherlands) | 90 mg |
| **Infusion** |  |
| Aminoplasmal (B. Braun, Germany) | 5 mL/h |
| Verapamil (Centrafarm, the Netherlands) | 0.25 mg/h |
